# Evidence-based cancer care: assessing guideline adherence of multidisciplinary tumor board recommendations for breast and colorectal cancer in a non-academic medical center

**DOI:** 10.1007/s00432-024-06049-x

**Published:** 2024-12-04

**Authors:** Carl-Stephan Leonhardt, Leopold Lanzenberger, Raphael Puehringer, Ulla Klaiber, Irene Hauser, Oliver Strobel, Gerald Prager, Martin Bodingbauer

**Affiliations:** 1 Department of Surgery, Landesklinikum Baden-Moedling, Baden, Austria; 2 Department of General Surgery, Medical University of Vienna, Waehringer Guertel 18-20, 1090 Vienna, Austria; 3 Usher Institute, University of Edinburgh, Edinburgh, UK; 4 Department of Internal Medicine, Landesklinikum Baden-Moedling, Baden, Austria; 5 Division of Oncology, Department of Internal Medicine I, Medical University of Vienna, Vienna, Austria

**Keywords:** Guideline, Guideline adherence, Hospitals, Community, Academic medical centers, Colorectal neoplasms, Breast neoplasms

## Abstract

**Purpose:**

Multidisciplinary tumor boards (MTB) are associated with improved outcomes. Yet, most patients in Western countries receive cancer care at non-academic medical centers. Guideline adherence of MTB recommendations in non-academic medical centers as well as factors contributing to non-adherence remain largely unexplored.

**Methods:**

This retrospective study followed the STROBE recommendations. All cases discussed at the MTB of the Landesklinikum Baden-Moedling, Austria, were eligible for inclusion. Guideline non-adherence was assessed by two reviewers independently using the AWMF S3 guidelines. Factors associated with guideline non-adherence were investigated using multivariable ordinal regression.

**Results:**

In total, 579 patients were included in the final analysis: 486 were female (83.9%) and 93 were male (16.1%), with a median age of 70 years (IQR 60–80). Most had breast cancer (n = 451; 77.9%), while 128 had colorectal cancer (22.1%). Complete adherence to guidelines was observed in 453 patients (78.2%), major deviations in 60 (10.4%), and minor deviations in 66 (11.4%) patients. Non-adherence was primarily due to patient preferences (n = 24; 40.0%), lack of surgical treatment recommendation (n = 24; 40.0%), and comorbidities (n = 9; 15.0%). After adjusting for relevant variables, predictors of non-adherence included older age at diagnosis (OR 1.02, 95% CI 1.00–1.04), colorectal cancer (OR 3.84, 95% CI 1.99–7.42), higher ECOG status (OR 1.59, 95% CI 1.18–2.16), and a more recent MTB conference (OR 1.20, 95% CI 1.03–1.41).

**Conclusion:**

Overall, guideline adherence was high for colorectal and breast cancer and comparable to results from academic medical centers. However, results need to be confirmed in other tumor entities.

**Supplementary Information:**

The online version contains supplementary material available at 10.1007/s00432-024-06049-x.

## Introduction

Multidisciplinary tumor boards (MTB) are flexibly composed panels comprising oncological, surgical, pathological, and radiological specialists as well as others tasked with the comprehensive review of individual cases and subsequent provision of a consented state-of-the-art treatment recommendation (Mano 2021; Walraven et al. 2019; Fehervari et al. 2021). MTBs have been associated with superior overall survival, diagnostic accuracy, quality of life, and patient satisfaction (Prades et al. 2015; Specchia et al. 2020; Freytag et al. 2020). Furthermore, both national and international guidelines, as well as certification requirements for cancer centers mandate the discussion of patients at MTBs (Wright et al. 2007; Basta et al. 2017; Borras et al. 2014).

Guideline adherence has been linked to improved outcomes in various tumor entities, and studies have investigated guideline adherence of MTB recommendations (Zhao et al. 2017; Thiels et al. 2019; Jaap et al. 2018; Worhunsky et al. 2015). However, most of these studies were conducted at academic medical centers, leaving guideline adherence at non-academic medical centers, including community hospitals, largely unexplored (Walter et al. 2023; Krause et al. 2022). Meanwhile, analysis of registry data indicates that patients treated in community cancer programs have a significantly lower likelihood of receiving guideline-compliant care (Thiels et al. 2019). This is particularly notable as most cancer patients in Western countries receive care at a non-academic medical center (Pfister et al. 2015; Wong et al. 2020). For instance, data from the United States indicate that approximately 80% of cancer patients are treated within a community hospital setting (The State of Cancer Care in America 2014).

Despite this fact and the association of guideline adherence with improved outcomes, there is a relevant gap in knowledge regarding the adherence of MTB recommendations in non-academic medical centers to established guidelines. Furthermore, potential factors contributing to guideline deviations and the extent to which MTB recommendations are implemented into clinical practice remain insufficiently understood. Importantly, academic medical centers typically have a variety of highly specialized MTBs for individual organ system, strongly contrasting the non-specialized MTBs commonly present at non-academic medical centers (Keating et al. 2012).

This exploratory study aims to assess the guideline adherence of MTB recommendations in a representative non-academic medical center in Austria. Of note, non-academic medical center/hospital is not a universally defined term and its definitions may vary across countries. In this study, non-academic medical center is considered a non-tertiary community hospital which is not part of a university hospital system. Additionally, this study aims to identify predictors of guideline deviation and to evaluate the implementation of guideline-adherent recommendations into clinical practice, thus addressing a critical gap in cancer care research.

## Material and methods

### Study design and study setting

This retrospective study adhered to the STROBE recommendations for observational studies (Elm et al. 2007). The study was approved by the local ethics committee (Vote GS3-EK-4/902–2024) and was conducted in accordance with the Declaration of Helsinki. All patients discussed at the MTB at the Landesklinikum Baden-Moedling, a non-academic medical center in Austria, were identified from a prospectively maintained electronic tumor board database (OIS, St. Poelten, Austria) and assessed for eligibility. The MTB at the Landesklinikum Baden-Moedling meets weekly in person and discusses a wide range of cases with malignancies involving different organ systems and specialties, including general surgery, oncology, urology, and others. Additionally, radiation therapists from other hospitals within the same healthcare network are joining the MTD online if needed.

### Study population

All patients with malignant tumors of the breast, colon, and rectum discussed at the MTB between 01/2019 and 03/2024 were eligible for inclusion. These entities were chosen since breast and colorectal cancer constitute the large majority of oncological cases treated and operated at the Landesklinikum Baden-Moedling. Only the first MTB discussion for each patient, during which a treatment recommendation was made, was considered. Patients with benign tumors, precancerous lesions, pregnant patients, as well as minors were excluded. Additionally, in line with the study’s objectives, patient records that lacked documentation of UICC/AJCC stage and/or TNM stage were excluded.

The following information was extracted from the electronic health record and the tumor board information system: date of birth, sex, ICD-10 diagnosis, date of first diagnosis, date of death, family history of cancer, TNM stage, UICC/AJCC stage, date of MTB conference, ECOG status, training level of the presenting physician (specialist vs resident), presenting department (surgery vs internal medicine), and MTB recommendation. Furthermore, data on the date of first therapy after MTB discussion, neoadjuvant treatment, hormone therapy, chemotherapy, immunotherapy, targeted therapy, number of treatment cycles, radiation therapy, and chemoradiation were also extracted.

### Primary and secondary outcomes

The primary outcome was guideline adherence of MTB recommendations. Secondary outcomes included the identification of predictors of guideline adherence, with odds ratios as estimands. Additionally, the study evaluated the implementation of MTB recommendations. Specifically, patients with guideline-adherent recommendations were analyzed to determine if the care they received was consistent with the MTB recommendation. These results are presented descriptively.

### Assessment of guideline adherence and deviations

Guideline adherence was classified as reported previously by Krause et al. (2022). Briefly, major criteria for guideline adherence were defined based on different treatment modalities with deviations in one or more criteria classified as major deviation. Minor criteria included specific therapeutic strategies within the respective treatment modalities. Deviation from one or more minor criterion was classified as minor deviation of guideline adherence (Krause et al. 2022). MTB recommendations lacking essential information were classified as non-assessable. This encompassed cases in which MBT recommendations suggested additional diagnostics, referral to other hospitals or specialists, renewed discussion at the MTB after an episode of watch-and- wait, as well as others.

Guideline adherence was assessed using the German AWMF guidelines (Arbeitsgemeinschaft der Wissenschaftlichen Medizinischen Fachgesellschaften—Associations of the Scientific Medical Societies in Germany) in their current version at the time of case presentation (Onkologie 2019, 2021). Two reviewers independently evaluated adherence to guidelines as well as reasons for any deviation, based on MTB protocols. In cases of disagreement, a discussion with the senior author was initiated until a consensus was reached. Interrater reliability was calculated empirically for the two initial reviewers using Cohen’s kappa (κ = 0.96, 0.93, and 0.90 for complete adherence, minor deviation, and major deviation, respectively); however, data analysis proceeded with the dataset after consensus was reached in discussion with the senior author.

### Statistical analysis

Demographic parameters and quantitative variables are described using means and standard deviations, while qualitative variables are reported as absolute and relative frequencies. Statistical inference was performed using two-tailed Student’s t-test, chi-square test, Fisher’s exact test, and Kolmogorov–Smirnov test. The normality assumption was investigated using the Shapiro–Wilk test.

Ordinal logistic regression was used to assess associations of selected variables with guideline adherence, minor deviation, and major deviation. Patients classified as non-assessable were excluded from the analysis. Given the low frequencies, ECOG 3 and 4 categories were combined. Additionally, due to the small number of patients presented by various specialties at the MTB, these specialties were grouped into surgical versus non-surgical categories. To account for the time of the MTB conference and potential changes over time, the earliest date of MTB conference in the dataset was set as zero, and all subsequent MTB conference dates were recalculated in years relative to this baseline. All variables were chosen based on domain knowledge and previous literature (Krause et al. 2022; Braulke et al. 2023; Hollunder et al. 2018).

Missing data for covariates were imputed for model building using imputation of multiples, under the assumption that data were missing at random (Buuren and Groothuis-Oudshoorn 2011). No imputation was performed for the outcome variable. Continuous variables were entered in the model and sensitivity analysis using non-linear forms did not lead to a relevant change in results. The proportional odds assumption for ordinal logistic regression was assessed using the Brant test (Brant 1990). Subgroup analysis was conducted only focusing on breast cancer and female sex, while keeping all the other variables in the model constant. Only female participants were included, as a very small number of male breast cancer cases were expected. A two-sided p-value < 0.05 was considered significant. The Bonferroni correction was applied to control the family-wise error rate when comparing characteristics of assessable versus non-assessable patients. All analyses were conducted using R (R project for Statistical Computing).

## Results

### Patient population

**Fig. 1:**
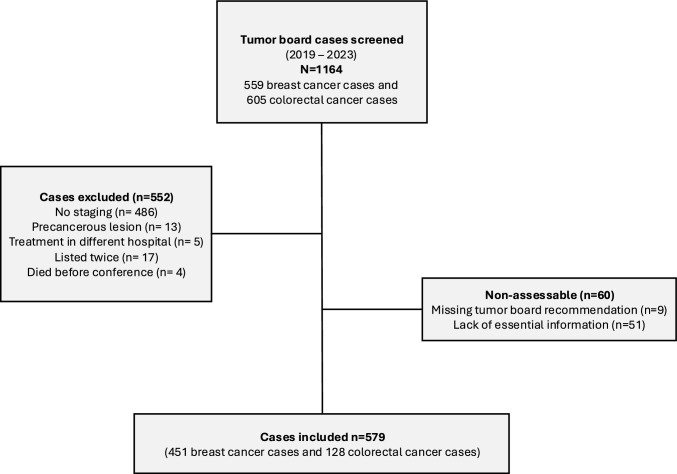
Flow-chart of the study population

After applying the exclusion criteria, a total of 639 patients remained. Of these, no guideline adherence could be assessed in 60 patients due to a lack of essential information in the MTB recommendation (n = 51) or no MTB recommendation (n = 9) (Fig. 1, *cf. methods*). The final cohort is depicted in Table 1. Most were females (n = 486, 83.9%) and the median age was 70 years (interquartile range 60–80). In total, 451 (77.9%) had breast cancer while 128 (22.1%) patients had colorectal cancer. Most MTB recommendations were adherent to guidelines (n = 453, 78.2%), while 60 (10.4%) exhibited major deviations, and 66 (11.4%) minor deviations. Major deviations were primarily lack of conservative treatment recommendation (n = 24; 40.0%), patient refusal of suggested treatment (n = 24; 40.0%), and patient-intrinsic factors including inoperability (n = 9; 15.0%) (Table S1).

**Table 1:** Characteristics of the study cohort

Patient characteristics	n	%
Total	579	100
Tumor entity		
Breast cancer	451	77.9
Colorectal cancer	128	22.1
Sex		
Male	93	16.1
Female	486	83.9
Age		
Median (IQR)	70.0 (60.0–80.0)	-
Positive family history		
Yes	106	18.3
No	227	39.2
Not available	246	42.5
ECOG status		
ECOG 0	412	71.2
ECOG 1	113	19.5
ECOG 2	35	6.0
ECOG 3	9	1.6
ECOG 4	4	0.7
Missing values	6	1.0
UICC stage		
UICC I	288	49.7
UICC II	145	25.0
UICC III	83	14.3
UICC IV	63	10.9

Treatment characteristics	n	%
Training level		
Resident	50	8.6
Specialist	457	78.9
Missing values	72	12.4
Department		
Surgery	537	92.7
Internal medicine	34	5.9
Missing values	8	1.4
Time MTB conference in years (mean, IQR)	2.76 (1.89–3.94)	**–**

### Predictors of guideline non-adherence

**Fig. 2:**
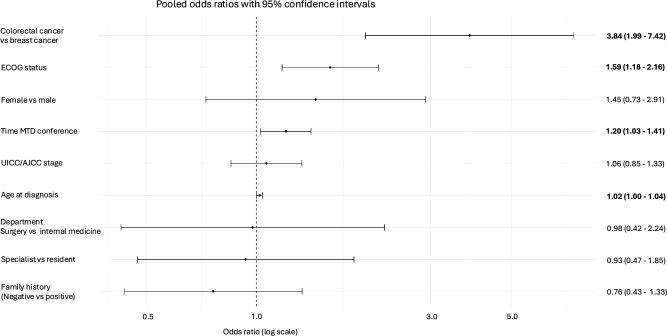
Forest plots with predictors of guideline deviation and associated odds ratios and 95% confidence intervals. Significant values are highlighted in bold

Univariable ordinal regression analysis revealed a significant association between age at diagnosis (OR 1.03, 95% CI 1.02–1.05), AJCC/UICC stage (OR 1.46, 95% CI 1.22–1.74), colorectal tumors (OR 3.12, 95% CI 2.05–4.75), and higher ECOG status (OR 1.89, 95% CI 1.50–2.39) and a higher probability of guideline deviation of the MTB recommendation. In contrast, female sex (OR 0.53, 95% CI 0.33–0.85) and presenting department surgery (OR 0.43, 95% CI 0.21–0.87) were associated with a lower probability (Table 2). After adjusting for prespecified variables, older age at diagnosis (OR 1.02, 95% CI 1.00–1.04), more recent MTB conference (OR 1.20, 95% CI 1.03–1.41), colorectal versus breast cancer (OR 3.84, 95% CI 1.99–7.42), and higher ECOG status (OR 1.59, 95% CI 1.18–2.16) were independently associated with a higher probability of guideline deviation (Fig. 2).

**Table 2:** Univariable ordinal logistic regression analysis

Variable	Odds ratio	95% Confidence interval	p value
Training level (specialist vs resident)	0.58	0.32–1.07	0.08
Age (continuous)	1.03	1.02–1.05	** < 0.001**
UICC (continuous)	1.46	1.22–1.74	** < 0.001**
Tumor type (colorectal vs breast cancer)	3.12	2.05–4.75	** < 0.001**
Department (surgery vs internal medicine)	0.43	0.21–0.87	**0.02**
Sex (female vs male)	0.53	0.33–0.85	**0.01**
ECOG	1.89	1.50–2.39	** < 0.001**
Family history (no vs yes)	0.88	0.50–1.55	0.65
Time MTB conference (continuous)	1.11	0.96–1.28	0.15

Significant values are shown in bold

### Subgroup analysis for breast cancer

**Fig. 3:**
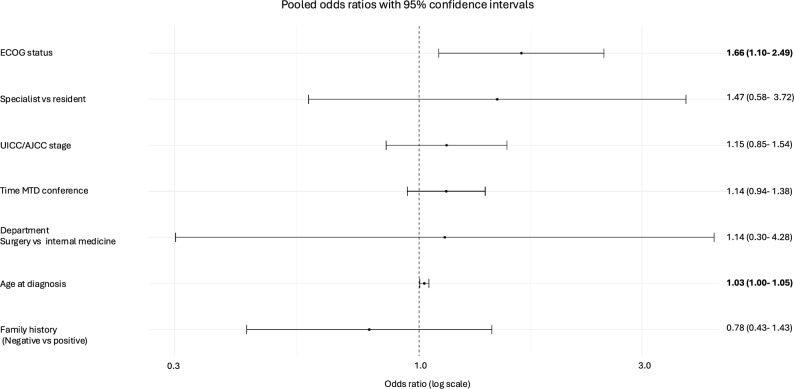
Forest plots with predictors of guideline deviation and associated odds ratios and 95% confidence intervals for breast cancer only. Significant values are highlighted in bold

As most patients were diagnosed with breast cancer, a subgroup analysis was conducted focusing on breast cancer only. Higher ECOG status (OR 1.66, 95% CI 1.10–2.49), and older age at diagnosis (OR 1.03, 95% CI 1.00–1.05) were significantly associated with a higher probability of guideline deviation of MTB recommendation (Fig. 3).

### Adherence to tumor board recommendation

Next, the implementation of guideline-adherent MTB recommendations into clinical practice was analyzed. While the implementation of the MTB recommendation could not be assessed in 22 (4.9%) patients, 395 (87.2%) were adherent to MTB recommendations. In contrast, 36 (7.9%) patients did not receive the recommended treatments.

## Discussion

Academic medical centers are integral to cancer care and research. However, most cancer patients receive treatment at non-academic medical centers within their local community. The present study revealed a high-level of guideline adherence of MTB recommendations at a representative non-academic medical center in Austria. Predictors of guideline non-adherence included older age at diagnosis, a higher ECOG status, colorectal cancer versus breast cancer, and a more recent MTB conference.

Adherence to guidelines has been associated with improved outcomes, and guideline adherence of MTB recommendations is considered a quality criterion for cancer care (Walraven et al. 2019). However, time pressure, a high number of cases, lack of MTB leadership, as well as lack of information including imaging, and pathologic workup, have been associated with low-quality decision making (Lamb et al. 2011a). On average, 3.2 min are spent on each case in MTBs (Lamb et al. 2011b). Interestingly, most literature on the quality of MTB recommendations originates from academic medical centers, while most patients receive treatment in non-academic medical centers (Wong et al. 2020). Furthermore, data indicate that increased travel time is associated with a worse quality of life, which potentially presents a barrier to the implementation of highly centralized care (Ambroggi et al. 2015; Bühn et al. 2020). Therefore, results from studies conducted in academic settings may not accurately reflect real-world experiences.

In the present study, guideline adherence was 78.2%, while studies from academic medical centers report adherence rates between 37 and 100% (Jaap et al. 2018; Walter et al. 2023; Krause et al. 2022; Brauer et al. 2017). However, differences in study designs, patient populations, guidelines, and others must be considered. Additionally, several predictors of guideline deviations were identified. Each additional year of age was associated with an approximately 2% higher probability of guideline deviation, while a one-point increase of ECOG status corresponded to a more than 60% higher probability of guideline deviation. Thus, both results indicate a clinically relevant difference. While performance status is frequently used as an indicator of a patient’s fitness for undergoing treatment, evidence suggests that it might be a suboptimal indicator (Sedrak et al. 2020). Additionally, randomized controlled trials, which form the foundation for guidelines typically include a highly selected population with low ECOG status, which might not reflect real-world patient cohorts (Booth and Tannock 2014). Our findings thus emphasize the lack of high-quality evidence for cancer treatment in the elderly (Sedrak et al. xxxx).

The strongest effect estimate was observed in patients suffering from colorectal cancer in contrast to breast cancer, resulting in a fivefold increase in the probability that the MTB recommendation would deviate from established guidelines. Several factors could be responsible for this finding. The Landesklinikum Baden-Moedling is certified as a breast cancer center by national authorities, potentially contributing to a higher guideline adherence in breast cancer cases. Interestingly, new study results, such as those from the RAPIDO trial in colorectal cancer, also led to guideline deviations, highlighting that deviations may be justified based on emerging evidence (Bahadoer et al. 2021).

Previous research from academic medical centers suggests that a higher tumor stage is associated with a higher probability of guideline non-adherence (Ronden et al. 2021; Hines et al. 2015). In our study, a higher UICC stage was associated with a 6% increased probability of guideline deviation. However, 95% confidence intervals were fairly evenly distributed around the null value. Future studies with a larger population are necessary to evaluate tumor stage as a predictor of guideline deviation.

Notably, a more recent case discussion at the MTB was associated with a higher probability of guideline deviation. The reasons for this are difficult to evaluate in a retrospective study; however, it is important to consider that the AWMF guidelines are not updated annually, while new evidence from clinical trials is continuously emerging. To address this, we opted for a relatively short eligibility time span when evaluating adherence, thereby reducing potential sources of heterogeneity. Interestingly, in the subgroup analysis focusing on breast cancer, only a higher ECOG status and older age at diagnosis remained independent predictors while a more recent MTB conference was not identified as a predictor.

Furthermore, previous reports have suggested that female sex is associated with guideline non-adherence of MTB recommendations (Walter et al. 2023). However, while significant in univariable analysis, after adjusting for potential confounding factors, no such association could be confirmed in our study. This might be due to different tumor entities.

Several strengths are worth mentioning. To the best of our knowledge, this is one of the first study specifically assessing guideline adherence of MTB recommendations and predictors of deviations in a non-academic medical center, thereby addressing a critical knowledge gap. Furthermore, a previously reported classification system was used to assess guideline adherence, enhancing validity and comparability of our results. Additionally, clinically relevant predictors of guideline non-adherence of MTB recommendations were identified using rigorous statistical methodology (Riley et al. 2022).

However, several caveats need to be acknowledged. Guideline adherence in this study was based on the AWMF guidelines, which are specific to German-speaking countries. In contrast, some guidelines, such as the NCCN guidelines, are updated more frequently and may better incorporate recent developments in the field. For instance, the S3 guideline for colorectal cancer was published in 2019, while the S3 guideline for breast cancer was last updated in 2021. This difference in the dates of the last updates might partially explain the higher probability of guideline non-adherence observed in colorectal cancer cases. Additionally, assessment of guideline adherence remains subjective. We tried to account for this by relying on the system reported by Krause et al. as well as requiring at least two reviewers to independently assess guideline adherence (Krause et al. 2022). Also, this study was performed in a Western European country with a universal healthcare coverage. Thus, the applicability of the results to other healthcare settings requires external validation.

Additionally, the present study only included colorectal and breast cancer cases, as these comprise the large majority of oncological surgeries at our hospital. Thus, the present results do not generalize to other malignancies, in particular cases of high-complexity oncological care such as esophageal and pancreatic cancer, for which a well-known volume-outcome relationship exists (Gooiker et al. 2011; Voeten et al. 2021).

Furthermore, no data on comorbidities, such as the Charlson Comorbidity Index (CCI), were available. This is noteworthy as previous studies have identified comorbidities as a relevant factor for guideline deviation (Krause et al. 2022). However, we tried to account for this limitation and incorporated performance status into our model, which has been shown to correlate with comorbidities (Grose et al. 2011). Also, as this was a retrospective study, selection bias cannot be ruled out. Only patients presented at the MTB were considered in the present study, no data were available on patients with breast or colorectal cancer who were not presented at the MTB. Additionally, no conclusions can be drawn on the effect of guideline adherent MTB recommendations on survival.

Finally, while this study took place at a non-academic medical center, it is part of an integrated care network. Thus, specialists at the MTB often join virtually from other centers. New possibilities in telemedicine might thus provide an opportunity for high-quality local cancer care. This is in line with data from the United States indicating higher guideline adherence at integrated network cancer programs compared to community cancer programs (Thiels et al. 2019). Some evidence points to improved outcomes after joining care networks with an academic-community hospital collaboration model (Tucker et al. 2020).

## Conclusions

In summary, the majority of MTB recommendations in a non-academic medical center are compliant with guidelines, indicating a reasonable quality of MTB recommendations. Furthermore, the identified predictors might aid in MTB decision-making. However, external validation is necessary, ideally in a prospective fashion. Additionally, investigation in other tumor entities besides colorectal and breast cancer should be conducted. Notably, complete adherence cannot and should not be ever reached as new evidence emerges and individualized patient care might not be adequately reflected in guidelines.

## Supplementary Information

Below is the link to the electronic supplementary material.

Supplementary file1 (DOCX 13 KB)

## Data Availability

No datasets were generated or analysed during the current study.
