# Supplementary material for: Evidence-based cancer care: assessing guideline adherence of multidisciplinary tumor board recommendations for breast and colorectal cancer in a non-academic medical center
Source: J Cancer Res Clin Oncol. 2024 Dec 4;151(1):4. doi: 10.1007/s00432-024-06049-x (PMC11618208; doi:10.1007/s00432-024-06049-x)
Supplement: Supplementary file 1 — Supplementary file1 (DOCX 13 KB) [file 432_2024_6049_MOESM1_ESM.docx]

**Supplementary Table 1:** Summary of factors for guideline deviations as well as non-assessable adherence.

| Major deviation (n=60) | n | % |
| --- | --- | --- |
| Patient refusal | 24 | 40.0 |
| Recommendation deviating from non-surgical treatment | 24 | 40.0 |
| Patient intrinsic factors | 9 | 15.0 |
| New study findings | 3 | 5.0 |
| Minor deviation (n=66) | **n** | **%** |
| Recommendation deviating from chemotherapy regimen | 29 | 43.9 |
| Best supportive care/follow-up | 14 | 21.2 |
| New study findings | 13 | 19.7 |
| Recommendation of additional therapy | 10 | 15.2 |
| Non-assessable adherence (n=60) | **n** | **%** |
| Lack of essential information | 51 | 85.0 |
| Missing tumor board recommendation | 9 | 15.0 |
